# Impact of fruit orientation and pelleting material on water uptake and germination performance in artificial substrate for sugar beet

**DOI:** 10.1371/journal.pone.0232875

**Published:** 2020-05-14

**Authors:** Sebastian Blunk, Jeroen Hoffer, Sandra Brosda, Martine I. de Heer, Craig J. Sturrock, Sacha J. Mooney

**Affiliations:** 1 Division of Agriculture and Environmental Science–University of Nottingham, Nottingham, England, United Kingdom; 2 Syngenta Seeds BV, Enkhuizen, The Netherlands; 3 Faculty of Medicine, The University of Queensland, Brisbane, Australia; 4 Syngenta Limited, Bracknell, England, United Kingdom; Brigham Young University, UNITED STATES

## Abstract

Water uptake into seeds is a fundamental prerequisite of germination and commonly influenced by commercial seed enhancement technologies. The effect of fruit orientation and contrasting pelleting materials on germination and biological performance of sugar beet was assessed. The results indicated there was orientation dependent fruit shrinkage of 37% for the operculum side supplied by moisture compared to 4% for the basal pore side. The expansion rate of 5% compared to the original size, which was also observed for non-shrinking seeds, indicated this was a temporary effect. This behaviour has importance for the application pelleting materials to seeds. Pellets composed of materials exhibiting low levels of swelling act as a water distribution layer which increased germination rates. Careful selection of pelleting material is crucial as it has direct implications on germination speed and subsequent establishment rates.

## 1 Introduction

**Fig 1:**
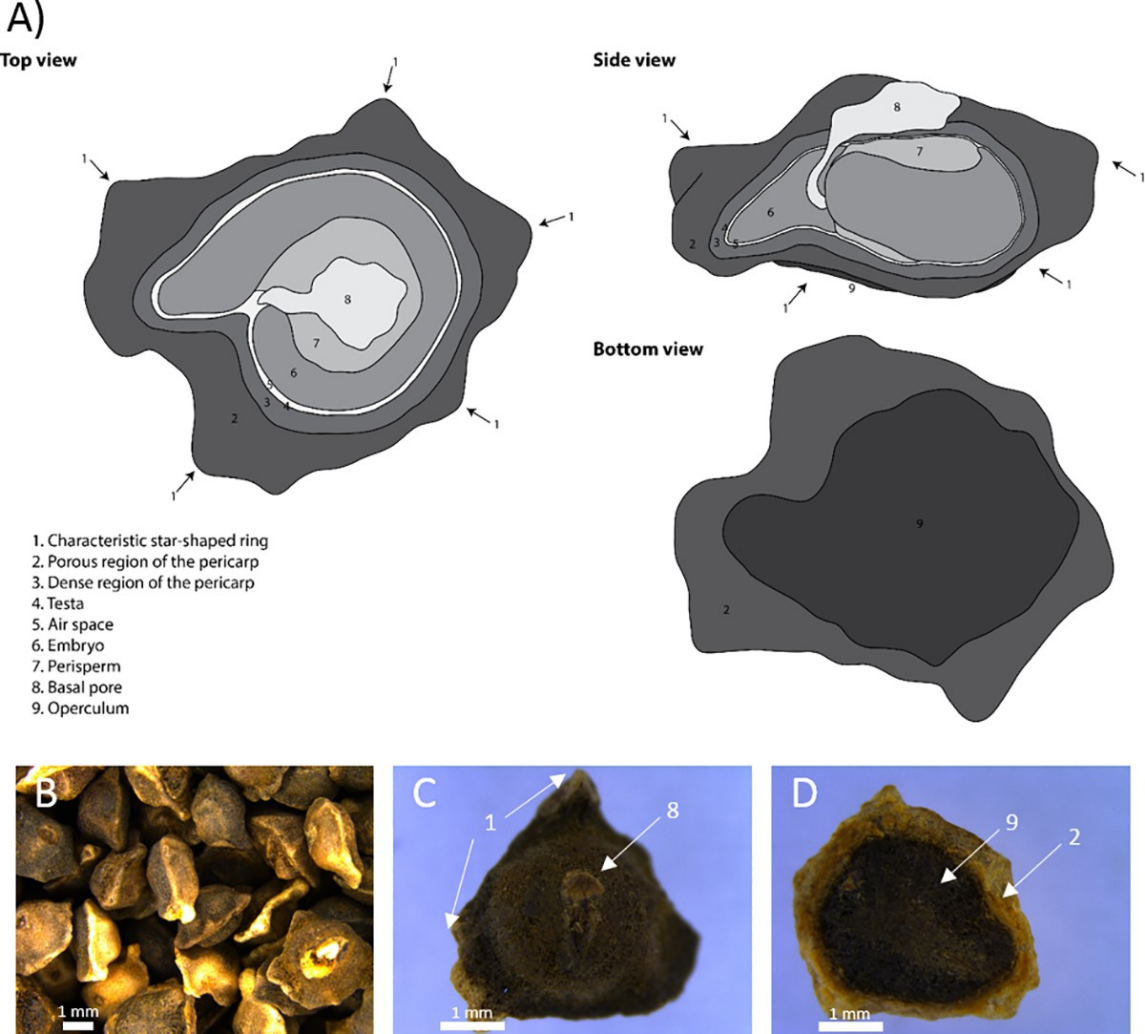
Schematic cross-section through a sugar beet fruit in three different orientations. A) (1) Star-shaped ring with 5 tips (2) Highly porous region in the pericarp (3) More firm region of the pericarp close to the true seed (4) Testa (5) Pore space between testa and embryo (6) Embryo made up radicle, hypocotyl and cotyledons (7) Perisperm with high starch contents (8) Basal pore (9) Operculum. B) Selection of naked fruits. C) Naked fruit with basal pore side upwards. D) Naked fruit with operculum side upwards.

When seeds enter a moist soil environment, the process of imbibition (i.e. water uptake) begins and germination is initiated upon contact of the dry seed surface to the moist soil matrix. For sugar beet, the true seed (embryo, perisperm and testa) is encapsulated in the pericarp, the fruit coat (dense sclerenchyma cells) containing the basal pore (loose cells) and the ovary cap (operculum) (Fig 1) [1].

Water uptake is proposed to be facilitated by the micropyle, a small hole allowing entry of water, the basal pore (former connection of the seed to the mother plant) and the operculum [2].

Plants are highly dependent on the available water in soil, which defined by water potential is often defined as that held between field capacity (ψ = -5 kPa (UK for a range of soil textures) after 24–48 h drainage) and permanent wilting point (no plant recovery beyond this point, ψ = -1.5 MPa) [3]. Plant establishment and development is severely impaired in soils when the matric potential is below the permanent wilting point which suggests this to be an appropriate lower limit for testing seed germination. However, some seeds (e.g. wheat) can germinate below the permanent wilting point [4–6]. The dry seeds of rape, wheat and corn have been reported to have a water potential as low as -400 MPa enabling the absorption of tightly bound water from soil pores and grains/aggregates [7]. Proteins present in the embryonic tissue are the driving force, having both negative and positive charges to attract polar charged water molecules [8]. Water attraction has previously been demonstrated using soybean (imbibing 2 to 5 times the dry weight mass) and corn (imbibing 1.5 to 2 times the dry weight mass) [9]. However, embryonic tissue has higher water binding properties than cotyledons, and therefore imbibes faster [10,11]. High starch content does not have an impact on imbibition rate unlike seed mucilage (cellulose and pectin containing) [8]. Seed mucilage is reported to improve water availability as mucilage swells upon water uptake and maintains water readily available [12]. As the seed has a more negative water potential compared to the surrounding soil, a rapid water flow towards the seed takes place which decreases over time due to the change in water potential within the seed. Therefore, a high soil water potential increases the germination percentage of seeds [13,14]. A species-specific threshold has to be reached to ensure radicle protrusion [14,15]. Hydraulic conductivity, the ease of water movement through pore space, typically differs by several orders of magnitude between field capacity and permanent wilting point [16]. Seed imbibition speed and germination percentage is therefore highly influenced with changes in soil hydraulic conductivity [13,17,18]. However, it has been reported that beyond 10 mm distance, most seeds do not attract water in most soils [8].

In soils, water availability is commonly negatively correlated to oxygen availability. In wet soils, poor aeriation may impede seed germination, whilst in dry soils it is not aeriation but a reduced water availability which will usually decrease germination. Reduced oxygen availability can be caused by severe rainfall events leading to the structural collapse of the seedbed followed by slumping [19] and soil compaction. Although poor aeriation increases carbon dioxide content, only the decrease in oxygen influences seed germination [20]. Hakansson *et al*. [21] confirmed in the models by Duval and Boiffin [22], Boiffin *et al*. [23] and Dürr *et al*. [24] indicating that soil structural collapse and following mechanical resistance had the highest impedance on seed germination. Aubertot *et al*. [25] found oxygen deficiency only occurred under rare circumstances for sugar beet. Hakansson [21] summarized how structural collapse of the seedbed followed by continuous wet conditions involving poor drainage and high oxygen consumption is one of the rare scenarios leading to oxygen deficiency for germination.

As seeds initiate the imbibition process from a dry quiescent state, water uptake occurs quickly to revitalize the metabolism of the seed [26]. The water uptake is initially controlled by permeation, and secondly, by growth [27,28]. The fruit coat represents a point of entry for water but also restricts the hydration rate to a certain degree as rapid water uptake may lead to cellular damage [29]. The general assumption is that seeds swell during the imbibition process which is followed by a shrinkage of the soil matrix due to water extraction, and also compaction near the seed surface induced by the swelling [30], although recent imaging assessments under laboratory conditions have shown this is not always the case [31]. The extent of swelling is determined by the presence of a pericarp as it swells faster in the initial stages of water uptake compared to embryonic sections, i.e. cotyledons [26,32]. Water diffusivity describes the control of the water flow by local conductivity and storage capacity [29]. Swelling behaviour has been modelled before using a seed-diffusivity water model [33] and the non-swelling model by Hadas and Russo [18] and Hadas [30].

Enhancement of water uptake potential may be facilitated by seed pelleting (originally developed for ease of planting) where powdered material is used to achieve a uniform geometry [34]. Seeds have been pelleted for many decades to ensure homogeneous germination through uniform sowing in germination trays (usually for vegetables) or in the field (e.g. sugar beet). Based on biological, chemical and physical characteristics, pelleting materials are chosen to either create homogeneous layers (fine materials, e.g. clay) or to increase size without significantly enhancing weight (coarse materials, e.g. woodmeal) or in a multi-material layer design. In the seed industry, pellet recipes are largely confidential, however, commonly used materials include clays (e.g. bentonite or attapulgite) [35–39], woodmeal [38], vermiculite [36,39], diatomaceous earth [37,40], talc [35,36,39], starch [37], kaolin, calcium carbonate, calcium oxide, fly ash, limestone, zeolite [39] or even leaf powder [41].

Powdered material is bound with water and a binding polymer, with varying amounts added based on the characteristics of the material. However, precise compositions are subjected to industrial confidentiality. Therefore, there is a gap of knowledge in the published literature on the effect of pelleting materials on seeds and their influence in water uptake behaviour. The aim of this study was to characterize the influence of common pelleting materials on the water uptake behaviour of seeds using sugar beet (*Beta vulgaris* L.) as a model species and to assess the influence of naked seed orientation and its swelling behaviour on this process. A selection of different, commonly used industrial pelleting materials was compared using germination tests and time-lapse imagery for the characterization of swelling behaviour to determine the influence of the pelleted material on water uptake rate and assistance during the period of germination. Such knowledge can underpin the development of future seed pellets supporting improved germination under suboptimal field conditions.

## 2 Materials & methods

### 2.1 Seed material and pre-treatments

Naked pre-treated sugar beet (*Beta vulgaris* L.) fruits (treatment name: naked, coded NK) of a single batch were supplied by Maribo Hilleshög, Landskrona, Sweden (Formerly: Syngenta Seeds AB, Landskrona, Sweden). A grading was performed prior to use but no chemicals were added to the fruit. The fruits were equilibrated under storage conditions with a temperature of 15°C and 30% relative humidity.

### 2.2 Pelleting treatments

Three materials for pelleting were selected based on diverging material properties: calcium bentonite (Fuller’s Earth, Sibelco BV) (treatment code: CB), diatomite (Celite 266, Imerys BV) (treatment code: DT) and woodmeal (C100, JRS GmbH Rettenmeier) (treatment code: WM). A 2% polymer solution (Polyvinylalcohol PVA Binder, BASF) was used as the binder. The pellet build-up was set to 100% weight increase. The protocol was aimed at fully encapsulating the seeds with the minimum amount of material required (the precise procedure is treated confidentially). The different biological, chemical and physical behaviour would have required adjusted amounts of glue which was accounted for by adjusting the water volume added to the pelleting mix. A comparable sample set was selected by sieving the seeds into a size class with a diameter of 3.25–3.75 mm.

### 2.3 Determination of the seed water content

The water content was calculated by dividing the mass of the oven-dried fruit by the mass of the wet or dry fruit. The determination of the fruit water content was conducted by oven drying 10 g of fruit in duplicates at 105°C (Termaks TS8056, Mettler AE163 / Mettler Toledo ME204).

### 2.4 Determination of water activity

The energy status of the water within a fruit is described by the water activity (a_w_) whereas the water content describes the amount of water present. The fruit water activity was determined using a Rotronic HygroPalm HP23-AWA handheld device with a HC2-PO5 sensor on a fruit lot at a room temperature of 15°C with 30% relative humidity (storage conditions).

### 2.5 Calculation of water potential (*ψ*)

The fruit water potential was calculated based on the measured water activity using the equation:

$$\psi =\frac{R*T}{M_{W}}ln(a_{w})$$

Where R is the universal gas constant (8.314 J / mol) and T, the temperature in degrees Kelvin (variable based on the measurement temperature). M_W_ is the molecular mass of water (18.01528).

### 2.6 Determination of swelling ratio

Fruit swelling (replication of 49 seeds on an 8 x 8 cm grid) was determined by time lapse photography with an 8 megapixel Raspberry Pi camera board V2 attached to a Raspberry Pi 1 model B+. Forty-nine fruits were placed in their specific orientations on top of two layers of germination paper (lower: Allpaper E2.82.82; upper: Anchor blue steel) and moistened with 12 ml water (150% water capacity). Evaporation was contained by a sealed lid. A Python script on the Raspberry Pi was set to record an image every 1 minute. The following time points were selected to reduce computational time: 0–30 min (1 min/ image); 30–60 min (3 min/image); 60–180 (5 min/image); 180–300 min (15 min/image); 300–480 min (30 min/image); 480–720 min (60 min/image); 720–1440 min (120 min/image); 1440–2880 min (360 min/image). Each photo was segmented to reveal the individual seeds and their respected swelling behaviour analyzed with Image J. The time-lapse photography was used to determine the swelling ratio (formula: area_timepoint_X_ / area_timepoint_0_) of different seed treatments over time. Seeds were germinated at 20°C day temperature with 16 hours of daylight and 16°C at night.

### 2.7 Germination test using PEG plates

The germination behaviour of the fruits pelleted with one of the three materials was assessed under different water potentials using PEG plates as described in [42]. A range of water potentials (-0.25 MPa; -0.50 MPa; -0.75 MPa; -1.00 MPa; -1.50 MPa) were selected by adding different concentrations of PEG8000. Seeds were germinated at 20°C day temperature with 16 hours of daylight and 16°C at night.

### 2.8 Germination paper test

Conventional paper-based germination tests were conducted using the same layout as for the time lapse photography and varying water volumes (adjusted based on mean water content of wet germination paper without gravimetric water loss) to simulate a variety of water potentials. Germination was tested in a paper test using different fractions (25%, 50%, 75%, 100% and 200%) of the water holding capacity (saturation of germination paper until no gravimetric water loss is detectable) of the paper. Seeds were germinated at 20°C day temperature with 16 hours of daylight and 16°C at night.

### 2.9 Definition of seed characteristics

The germination percentage was plotted against the time after sowing. Total germination refers to the time point after which all fruits germinated. The inflection point is reached at the time point where half of the fruits germinated as determined by fitting a sigmoidal 4P Hill Model to the germination curve [43].

### 2.10 Statistical analysis

An automated image processing pipeline for 2D time-lapse imaging was developed using ImageJ and R for image pre-processing and data analysis. The resulting data was analyzed using a full factorial ANOVA. Differences in germination behaviour of fruit treatments under different water availability were assessed using a full factorial ANOVA. All error bars represent standard error of the mean or pooled standard error where appropriate.

## 3 Results

### 3.1 Fruit swelling in untreated fruits

**Fig 2:**
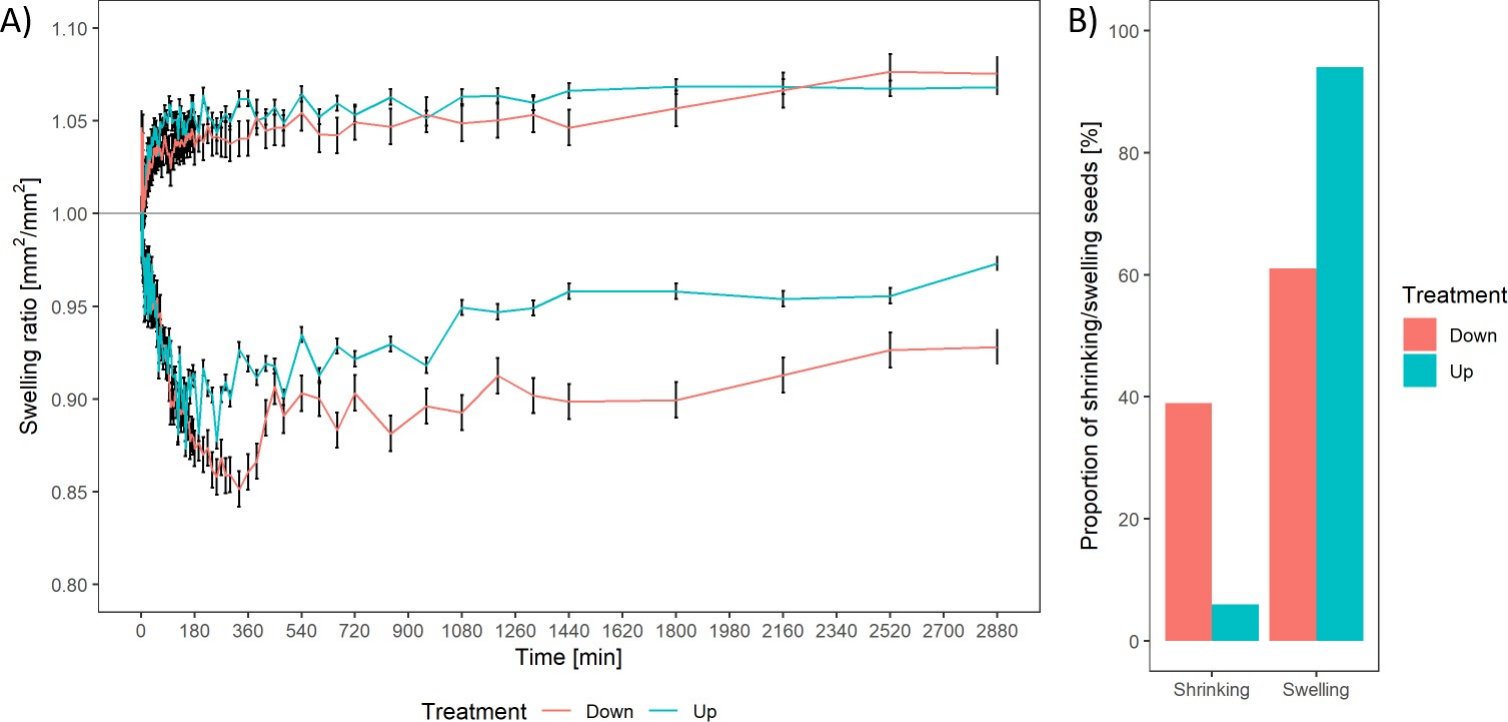
Display of the swelling behaviour of the imaged fruits as a ratio. A) Fruits were separated into two groups based on whether the majority of time points showed a swelling or shrinking behavior categorized by a swelling rate above or below 1. B) The proportion of fruits shrinking or swelling in relation to its orientation is displayed.

An increase of 5% in 2D surface area was observed for the majority of the fruits over the imaging period of 2 days with a comparable area of the seed surface touching the germination paper. Around 37% of the naked fruits with the operculum side touching the wet surface shrunk (more than 50% of the time points exhibited a swelling rate below 1 in comparison of the current time point with the dry state) to different extents immediately upon water uptake before swelling commenced. In comparison, only 6% of the naked fruits with the basal pore side touching the wet surface exhibited this behaviour. In general, shrinkage was observed during the first 6 hours of water uptake and replaced by swelling for the later imbibition stages. For the naked treatments, an increase of 5% or decrease of 10–15% of the original dry area was observed. Interestingly, the 5% increase in area was also observed during the later time points for the fruits exhibiting the shrinkage initially. However, even after the swelling period, the fruits exhibiting an initial shrinking did not regain their original size before germination (Fig 2).

### 3.2 Influence of fruit orientation on germination rate

**Fig 3:**
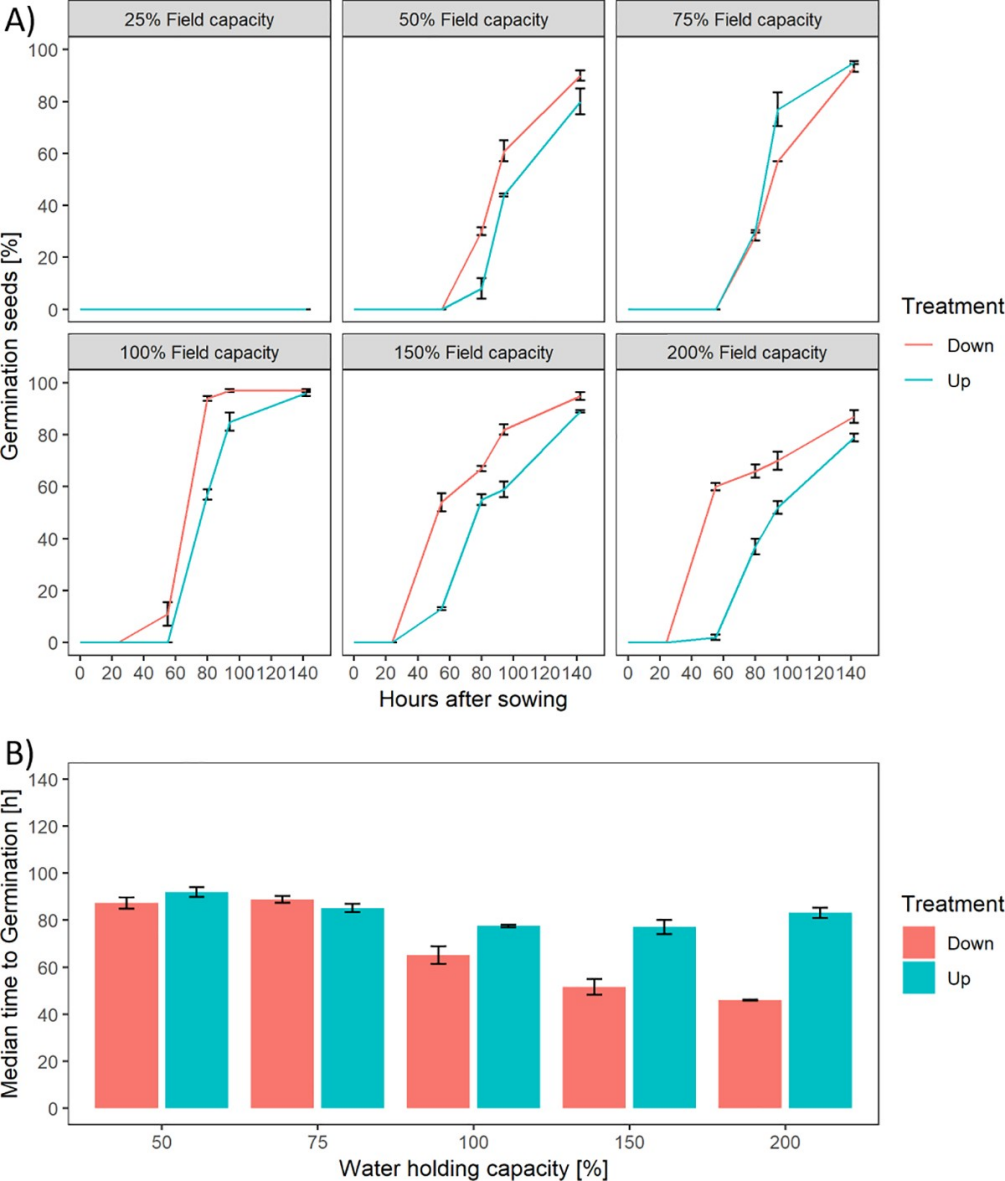
Test of sugar beet fruit germination on germination paper using different levels of available water. A) Percentage of germination over time shown per level of available water. B) Duration until inflection point is reached per level of available water.

The germination test showed a faster germination rate if the operculum side of the fruit was touching the moist germination paper compared to the basal pore side of the fruit, however a similar total germination was observed in both situations (Fig 3). After early time points, increasing levels of moisture (up to 200% water holding capacity) was more beneficial for operculum orientated germination compared to 100% water holding capacity. This changed after 3–4 days of growth with a higher germination rate for 100% water holding capacity. Throughout the time series, the operculum side treatment exhibited a higher level of germination except at 75% water holding capacity. Overall, the level of available water had a significantly positive effect on total germination (p = 0.012). The orientation had no significant effect on total germination (p = 0.763), however a significant effect on the inflection point was detected (p < 0.001). The interaction of available water and orientation was significant (p < 0.001).

### 3.3 Characterization of fruit treatments

**Fig 4:**
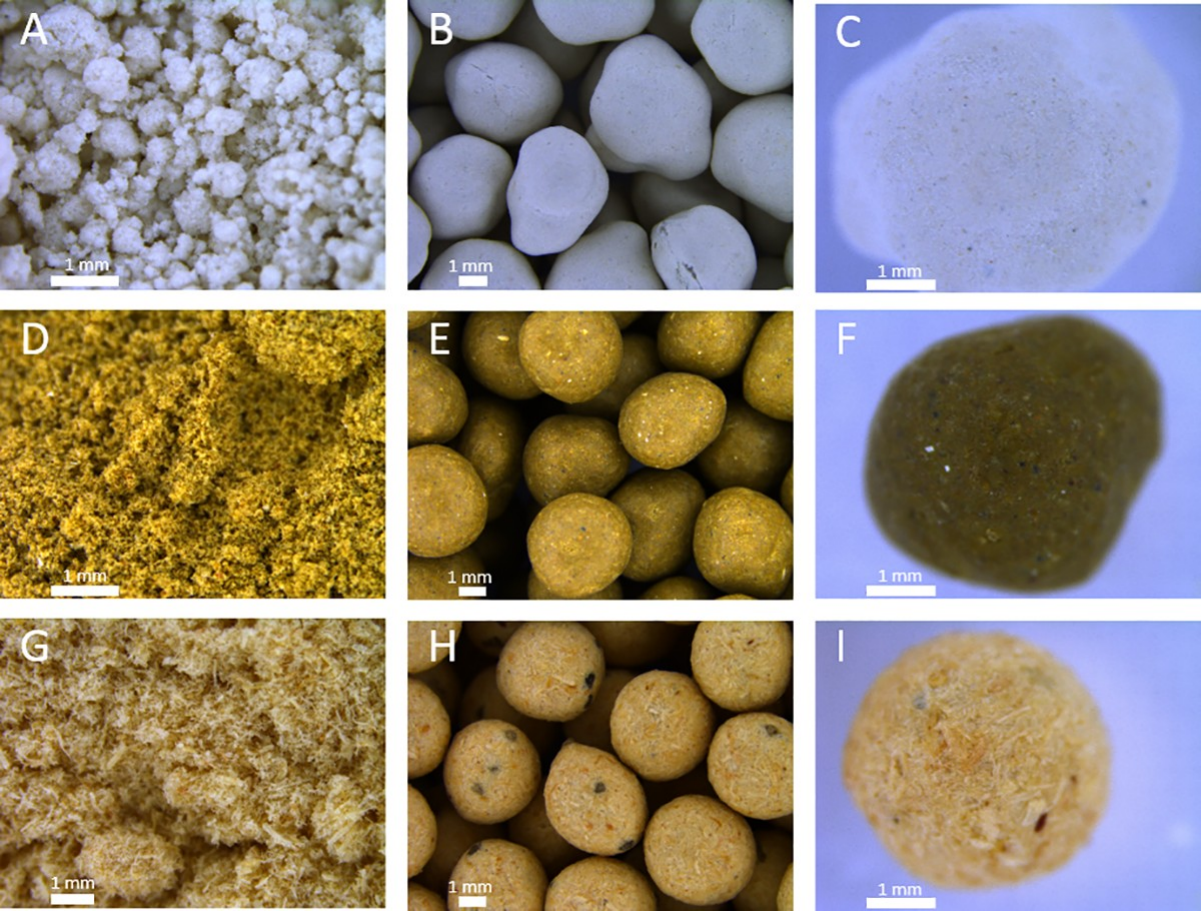
Light microscopy of pelleting treatments. A) Diatomite powder. B) Diatomite pelleted fruits in a batch. C) Single diatomite pelleted fruit. D) Calcium bentonite powder. E) Calcium bentonite pelleted fruits in a batch. F) Single calcium bentonite pelleted fruit. G) Woodmeal C100 fibers. H) Woodmeal pelleted fruits in a batch. I) Single woodmeal pelleted fruits.

Pelleting procedures were adjusted based on the material properties to create an economically viable round-shaped fruit (Fig 4). The 1000 fruit weight for the pelleting treatments showed significant differences (p < 0.001) between the treatments (DT: 38.23 g (± 0.18 g); CB: 52.38 g (± 0.18 g); WM: 20.44 g (± 0.26 g)).

**Fig 5:**
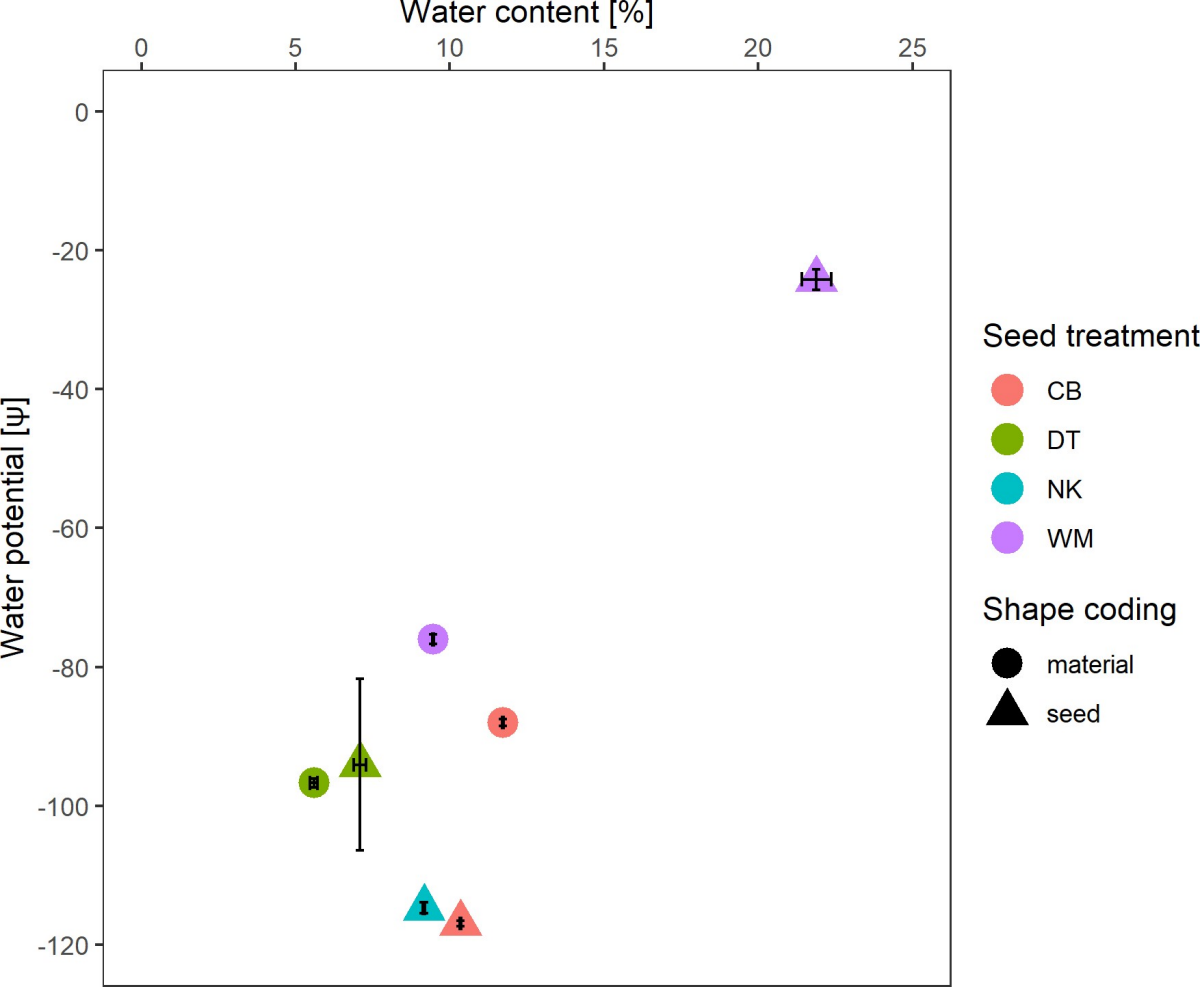
Water potential and water content of all fruit treatments. Water potential calculated based on temperature and water activity measurement taken comparing the plain material powder and the seed treatments. Error bars represent standard error of the mean.

The data showed a high water content and lower water potential for the woodmeal treatments which can be explained chemically by hydroxyl group bonds forcing water inwards between microfibrils causing swelling (Fig 5).

**Fig 6:**
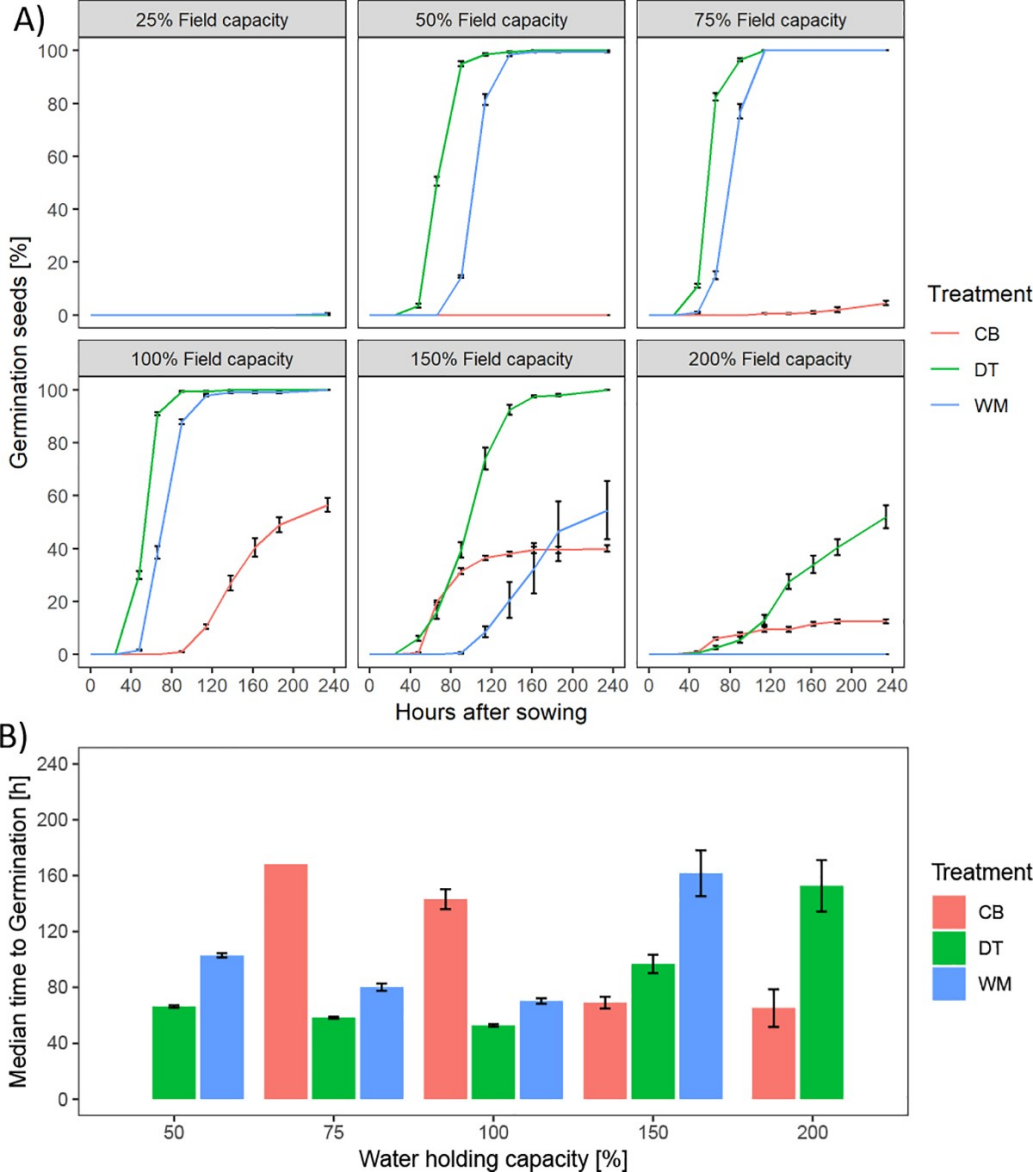
Germination paper test of pelleting treatments. A) Germination percentage over time separated by available moisture. B) Time until inflection point reached. CB = Calcium bentonite, DT = Diatomite, WM = Woodmeal.

The other treatments exhibited similar water potential and slight differences in moisture content. The fastest germination under the lowest water availability was exhibited by the DT treatment followed by WM (Fig 6).

**Fig 7:**
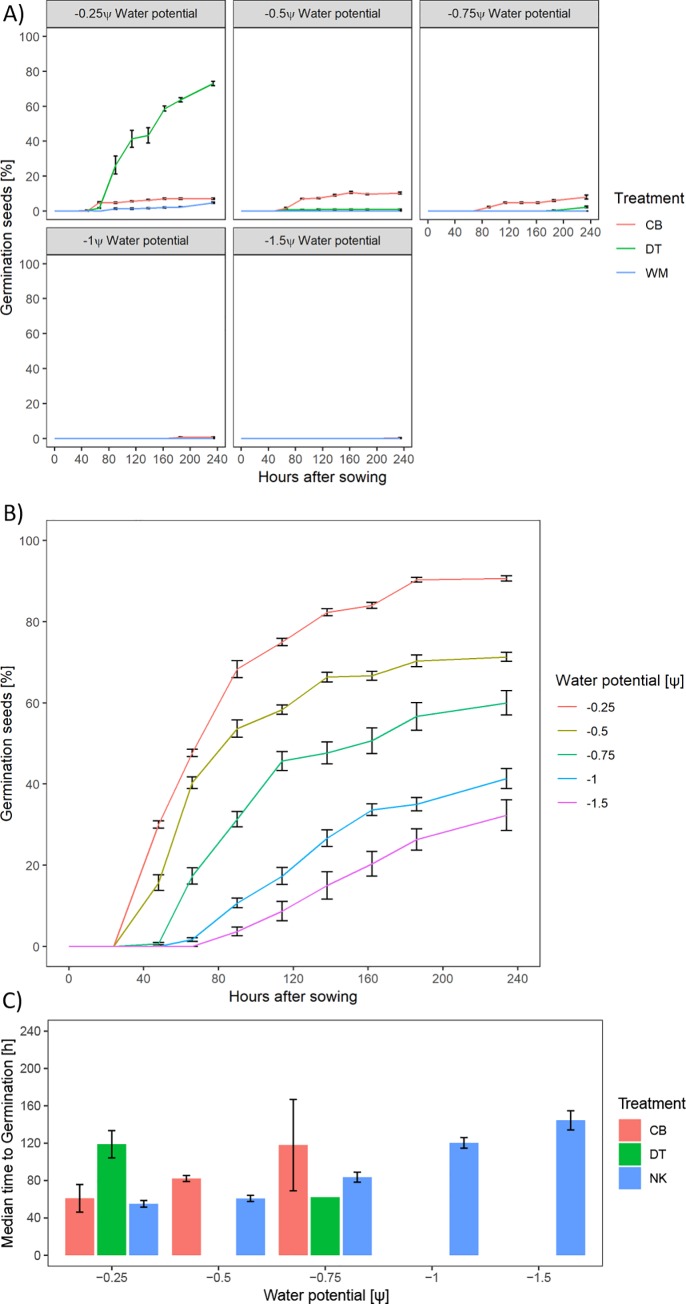
Germination test of pelleting treatments on PEG plates. A) Germination percentage over time separated by water potential. B) Separate display of the naked treatment under varying water potential. C) Median time to germination separated by water potential.

Very low germination rates were found for the CB treatment in all water levels compared to DT and WM. A significant interaction between available water and treatment was found for the speed of germination which is due to the effect on germination posed by the pelleting materials. A separate analysis of available water showed a significant effect for DT and WM (p = 0.003) as well as for CB (p < 0.001). A significant effect on total germination at increasing moisture availability was found (p < 0.001). Treatments WM and CB showed almost no germination for all applied water potentials (Fig 7A).

**Fig 8:**
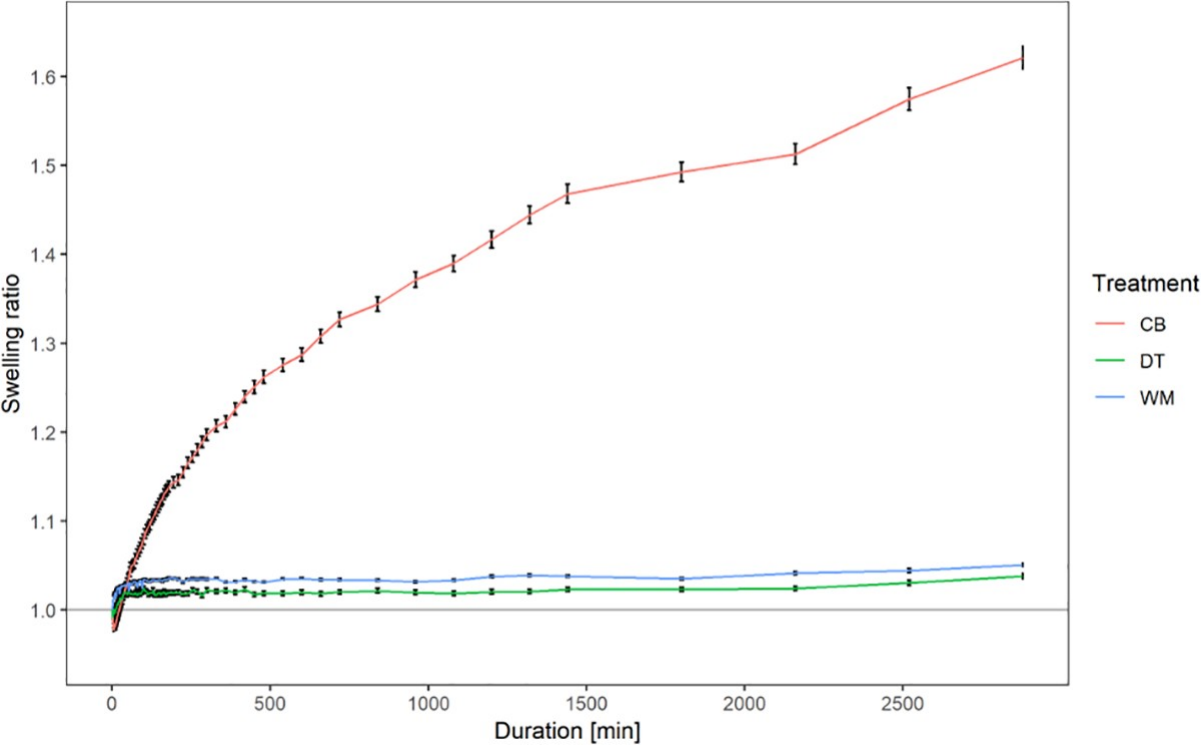
Swelling ratio of pelleted treatments imaged over two days. CB = Calcium bentonite; DT = Diatomite; WM = Woodmeal.

Compared to the naked treatment, DT had a reduced germination rate under 100% water holding capacity. Significant effects for treatment and applied water potential on inflection point were noted. Water potential exhibited a significant negative effect on germination. The naked treatment showed a significant effect (p < 0.001) of water potential over time (Fig 8).

### 3.4 Fruit swelling in pelleted fruits

The swelling behaviour of pelleted fruit treatments was observed as an indicator for the amount of water absorbed over time assessed similar to the naked seeds. The diatomite and woodmeal treatments exhibited very little swelling resulting in a similar swelling pattern compared to the naked treatments. The calcium bentonite treatment however showed a significant increase in area of up to 1.7 of the original size throughout the measurement period although there was large variability. The pelleting treatments generally masked swelling or shrinking behaviour of the encapsulated fruit, however 2% (1/49 fruits) for CB, 16% (8/49 fruits) for DT and 0% (0/49) for WM exhibited a shrinking behaviour in more than 50% of the time points.

## 4 Discussion

Time-lapse photography enabled the observation of changes in individual fruits in 2D over time with regards to seed surface area. Testing the orientation of the sugar beet fruit on moist germination paper using different amounts of water revealed no significant effect on total germination, however the speed of germination was significantly affected. A shorter amount of time was needed until the inflection point was reached for the operculum touching the moist surface, indicating the basal pore does not represent the primary way of water uptake. This contradicts a common assumption that the basal pore is one of the major entry points of water uptake besides the micropyle as the pericarp mainly consists of an impervious layer of sclerenchyma cells [44]. However, as the total germination reaches similar levels, this indicated water was distributed from the basal pore throughout the pericarp towards the main areas of water uptake. This was mostly apparent under high water availability suggesting low amounts of water equilibrate within the pericarp slowly before reaching the embryo whereas high amounts moisten the dry pericarp tissue and therefore imbibe the embryo more quickly. The sclerenchyma (inner layer) and parenchyma (outer layer) cells are sensitive to oxygen availability, which can be problematic when the fruit is placed one the filter paper one-sided. It is possible a water film surrounding the fruit might impair oxygen availability similar to a water-logged state described for soil and thereby also affecting germination rate although this would only affect one side of the seed [21]. The present results might indicate the basal pore site is more sensitive to reduced oxygen availability compared to the operculum site.

Additionally, the time-lapse observation of imbibing fruits revealed shrinkage of naked fruits upon water uptake during early time points. A stronger effect of shrinkage was detected for the operculum side touching the wet surface which correlates with the faster germination rate observed in the paper test. Chemically, water molecules push apart microfibrils causing the uniform swelling behaviour in all seed treatments. Upon water intake, the water dissolves sugars and polysaccharides, compound crystals as well as germination inhibiting crystals in the pericarp tissue [45]. This dissolution may lead to a structural collapse of the porous structure resulting in shrinkage when observed from above. This was less common for fruits with the basal pore touching the wet surface which might indicate a non-uniform distribution of germination inhibiting crystals within the pericarp with a concentration in the operculum between individual fruits [46]. Further research is necessary to probe the dominance of the contrasting mechanism under different conditions.

The application of pelleting material aims to create a uniform surface through which water is supposed to be distributed equally. In theory, this would significantly reduce the effect of fruit orientation in soil. Comparison of water potential and water content of the pelleting treatments indicated WM to exhibit the lowest pull on surrounding water sources whereas CB and DT exhibited similar forces as NK. This is most likely due to the manufacturing process as the production of WM pellets requires large amounts of liquid therefore increasing the initial moisture content. Whilst the CB treatment is highly durable, the WM treatment is fragile to the touch in the presence of water and the DT treatment loses integrity even without physical influence. The low levels of swelling for both WM and DT treatments, which was mainly driven by the inherent swelling behaviour of the fruit, indicates that the material itself has a low water holding capacity and acts in a distribution capacity. The CB treatment in comparison served as both a distributor and a storage organ indicated by high levels of swelling. As CB exhibited the poorest germination efficiency under most water availabilities (except for 200% water holding capacity), this would indicate that permeability is a more important trait than water holding capacity in this case or that this specific material does retain water stronger than the fruit is able to absorb and therefore delays germination. DT exhibited the most consistent and highest levels of germination under varying water availability indicating that the physicochemical properties of the material allow a better trade-off of distribution and water holding capacity compared to WM. WM showed a higher water retention in a funnel material test due to its spongy structure and percolation indicated a higher porosity in comparison to DT (S2 Fig in S1 File). As low soil water availability is a common agricultural problem, an intermediate material that is able to store water (i.e. exhibits a small degree of swelling) and distributes water to similar degrees as DT would be beneficial. DT may therefore provide water to the germinating seed in quantities that support germination and improve seedling establishment.

The discrepancy between the traditional paper test and the PEG agar setup might be explained by the difference in oxygen availability. However, [47] and [48] found that oxygen availability is not limiting in a PEG agar setup. Whilst the filter paper test represents the traditional germination test, the PEG agar is more representative of soil conditions, in the sense that seeds are completely encapsulated by the medium with limited actual contact points. The fact that filter paper tests are closer to an ideal situation for germination was apparent from the quicker germination compared to PEG media.The shrinkage effect described for naked fruits also translated to the pelleted treatments. Upon shrinkage of the fruit itself, a partial vacuum may be created between the fruit surface and the pellet material which lead to a reduction in surface area. Another explanation could be the presence of the binding substance which consolidates the pelleting material upon shrinkage.

## 5 Conclusions

The shrinkage behaviour of sugar beet fruits is significantly influenced by fruit orientation. However, this does not appear to impact on total germination under varying water availability conditions, though it could give an indication for why natural variability can be high in the field. The selection of pelleting material has a significant impact on germination. Here it was shown that the water distributing material, DT, exhibited higher levels of germination under most water availabilities than the water absorbing material, BC. Further studies concerning on shrinkage behaviour of fruits are needed to understand potential effects on germination and the quality of seedling establishment. Thorough pre-sowing non-destructive imaging of individual fruits could lead to the identification of markers to pre-sort fruit lots prior to fruit processing which would ultimately support efforts to maximize yield and efficiency which are urgently needed.

## Supporting information

S1 File Supplementary methods and results. (DOCX)
