# Supplementary material for: Impact of fruit orientation and pelleting material on water uptake and germination performance in artificial substrate for sugar beet
Source: PLoS One. 2020 May 14;15(5):e0232875. doi: 10.1371/journal.pone.0232875 (PMC7224476; doi:10.1371/journal.pone.0232875)
Supplement: S1 File — (DOCX) [file pone.0232875.s001.docx]

# Supplementary methods

## Imbibition curve

An imbibition curve was determined for NK by imbibing meshed bags of 10 g of fruits in water. The precise weight was determined after selected time intervals (centrifugation of meshed bags prior to weight determination to remove excess of water for 1 minute at 2800 rpm; AEG Electrolux SV4028). No imbibition curve was determined for the pelleted treatments due to the low water stability of the materials which would result in inaccurate gravimetrical measurements.

## Conductivity and retention test

A layer of approximately 2-3 cm height made of pelleting material (30 g) was placed on top of a 40 – 100 µm^2^ membrane in a funnel (Robu 116, 500 ml) after oversaturating the material powder with 500 ml water. The notional field capacity of the material was determined gravimetrically after water dripping ceased representing the water retention of the material. The pellet layer was then dried in an oven at 105°C overnight. An infiltration rate was determined using a burette by measuring the duration of dripping water (flow rate adjusted to the material) in relation to the material height and density. Conductivity was determined with continuous dripping and determination of the flow through volume using the burette with a volume of 100 ml of water. The burette flow rate was adjusted separately to avoid the formation of a water layer on top of the pellet material. A ratio of the conductivity and the flow rate was then calculated for comparability.

# Supplementary Results

**S1 Fig: Imbibition curve of naked treatments**. Imbibition curve was determined in a water bath over time using pre-determined weights


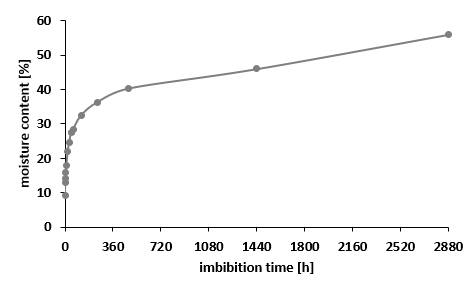


## Characterization of pelleting materials

Three common pelleting materials were selected based on the biological, chemical and physical properties. Significant differences in water content of the powder were determined initially with 11.72% (± 0.02%) for CC, 5.59% (± 0.12%) for DT and 9.46% (± 0.03%). Furthermore, significant differences in water potential were detected with -88.01 MPa (± 0.46 MPa) for CC, -96.72 MPa (± 0.65 MPa) for DT and -76 MPa (± 0.71 MPa). Conductivity and retention capabilities tested in a funnel system (Supplementary Figure 2).

**S2 Fig: Funnel system to test retention and conductivity of pelleting materials**. A burette is dripping water on an oven dried layer of pelleting material. The time needed for imbibition and conductivity is measured and calculated as a rate.


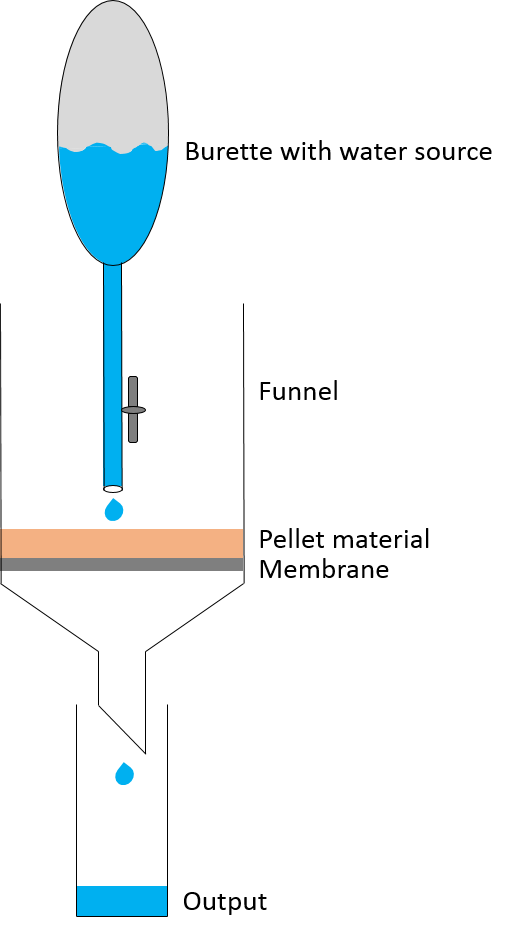


Testing of WM (reaching field capacity after about 2 hours) and DT (reaching field capacity after two days) was feasible while CB did not reach field capacity after one week (90 ml of prior added 500 ml drained). The woodmeal exhibited a moisture content of 465.68% at field capacity compared to 60.13% tested with diatomite. The resulting layer height differed significantly with an average height of 2.58 cm for the WM layer (volume: 146.59 mm^3^; density: 0.18 g/mm^3^) and 0.93 cm for the DT layer (volume: 51.07 mm^3^; density: 0.55 g/mm^3^). After oven drying, the layer shrank to an average height of 1.96 cm (volume: 119.16 mm^3^; density: 0.22 g/mm^3^) and 0.75 cm (volume: 45.40 mm^3^; density: 0.62 g/mm^3^), respectively. The burette flow rate was set to the highest flow rate without creating a water layer on the surface resulting in an infiltration rate of 0.167 ml/g/min for WM and 0.017 ml/g/min for DT. The penetration rate of WM was determined 0.362 (ratio of flow rate and leakage rate) for the first run immediately after infiltration of the dry layer. The percolation rate increased and to 0.985 after four runs. For DT, the percolation rate was 0.226 initially and increased in a similar way during wetting of the material.
